# Branched-chain amino acids in health and disease: metabolism, alterations in blood plasma, and as supplements

**DOI:** 10.1186/s12986-018-0271-1

**Published:** 2018-05-03

**Authors:** Milan Holeček

**Affiliations:** Department of Physiology, Faculty of Medicine in Hradec Kralove, Charles University, Simkova 870, 500 03, Hradec Kralove, Czech Republic

**Keywords:** Cachexia, Ammonia, Glutamine, Diabetes, Cirrhosis, Nutrition

## Abstract

Branched-chain amino acids (BCAAs; valine, leucine, and isoleucine) are essential amino acids with protein anabolic properties, which have been studied in a number of muscle wasting disorders for more than 50 years. However, until today, there is no consensus regarding their therapeutic effectiveness.

In the article is demonstrated that the crucial roles in BCAA metabolism play: (i) skeletal muscle as the initial site of BCAA catabolism accompanied with the release of alanine and glutamine to the blood; (ii) activity of branched-chain keto acid dehydrogenase (BCKD); and (iii) amination of branched-chain keto acids (BCKAs) to BCAAs. Enhanced consumption of BCAA for ammonia detoxification to glutamine in muscles is the cause of decreased BCAA levels in liver cirrhosis and urea cycle disorders. Increased BCKD activity is responsible for enhanced oxidation of BCAA in chronic renal failure, trauma, burn, sepsis, cancer, phenylbutyrate-treated subjects, and during exercise. Decreased BCKD activity is the main cause of increased BCAA levels and BCKAs in maple syrup urine disease, and plays a role in increased BCAA levels in diabetes type 2 and obesity. Increased BCAA concentrations during brief starvation and type 1 diabetes are explained by amination of BCKAs in visceral tissues and decreased uptake of BCAA by muscles.

The studies indicate beneficial effects of BCAAs and BCKAs in therapy of chronic renal failure. New therapeutic strategies should be developed to enhance effectiveness and avoid adverse effects of BCAA on ammonia production in subjects with liver cirrhosis and urea cycle disorders. Further studies are needed to elucidate the effects of BCAA supplementation in burn, trauma, sepsis, cancer and exercise. Whether increased BCAA levels only markers are or also contribute to insulin resistance should be known before the decision is taken regarding their suitability in obese subjects and patients with type 2 diabetes.

It is concluded that alterations in BCAA metabolism have been found common in a number of disease states and careful studies are needed to elucidate their therapeutic effectiveness in most indications.

## Background

The branched-chain amino acids (BCAAs), valine, leucine, and isoleucine are essential amino acids, which have been studied in a number of disorders, notably liver cirrhosis, renal failure, sepsis, trauma, burn injury, and cancer. BCAA supplementation has been thought to promote anabolic pathways and therefore mitigate cachexia, prevent or treat signs of hepatic encephalopathy, attenuate fatigue during exercise, promote wound healing, and stimulate insulin production. However, until today, there is not consensus regarding their use as nutritional supplements [1, 2].

The intentions of this article are to: (i) review main metabolic pathways and supposed effects of BCAAs; (ii) assess the causes of alterations in metabolism and BCAA levels in various healthy and pathological conditions; and (iii) provide current views on their use as nutritional supplements for the main possible indications. As the main pathways of all three BCAAs are common and mixtures of all three BCAAs are used in most indications, the article does not describe the differences in effects of specific BCAAs.

## BCAA metabolism

### BCAA catabolism (Fig. 1)

Unlike most amino acids, the initial step of BCAA catabolism does not take place in the liver due to low hepatic activity of branched-chain-amino-acid aminotransferase (BCAT), the first enzyme in the BCAA catabolism pathway. Therefore, the BCAA increase rapidly in systemic circulation after protein intake and are readily available to extrahepatic tissues. This phenomenon gives a unique advantage to the BCAA-based nutritional formulas compared with others, especially those targeted on muscles and brain.

**Fig. 1:**
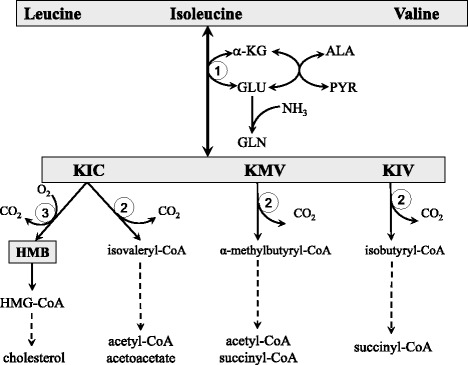
Main pathways of BCAA catabolism. ALA, alanine; GLU, glutamate; GLN, glutamine; HMB, β-hydroxy-β-methylbutyrate; HMG-CoA, 3-hydroxy-3-methyl-glutaryl-CoA; KIC, α-ketoisocaproate (ketoleucine); KIV, α-ketoisovalerate (ketovaline); KMV, α-keto-β-methylvalerate (ketoisoleucine); α-KG, α-ketoglutarate. 1, branched-chain-amino-acid aminotransferase (BCAT); 2, branched-chain α-keto acid dehydrogenase (BCKD); 3, KIC dioxygenase

The initial site of most of the BCAA catabolism is skeletal muscle because of the BCAT high activity. The BCAT reaction involves the reversible transfer of the BCAA amino group to α-ketoglutarate (α-KG) to form glutamate and the corresponding branched-chain keto acids (BCKAs), α-ketoisocaproate (KIC, ketoleucine), α-keto-β-methylvalerate (KMV, ketoisoleucine), and α-ketoisovalerate (KIV, ketovaline). Glutamate then acts as an amino group source to form alanine (ALA) from pyruvate or as a substrate for ammonia detoxification to glutamine (GLN). GLN, ALA, and a significant portion of the BCKA are released from muscles to the blood.

The second enzyme of BCAA catabolism, branched-chain α-keto acid dehydrogenase (BCKD), is a multienzyme complex located on the inner surface of the inner mitochondrial membrane, which catalyzes irreversible decarboxylation of the BCKA to the corresponding branched-chain acyl-CoA esters. The BCKD is regulated by the phosphorylation-dephosphorylation mechanism. Phosphorylation mediated by a specific kinase results in inactivation, while dephosphorylation by a specific phosphatase activates the enzyme. Changes in kinase activity may play a main role.

**Fig. 2:**
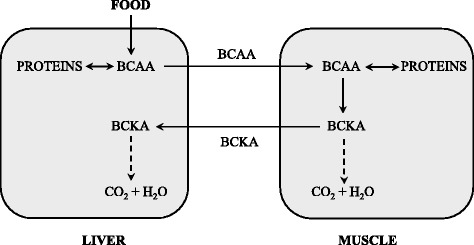
Cooperation of the muscles and the liver in BCAA catabolism. BCAA, branched-chain amino acids; BCKA, branched-chain keto acids

The BCKD activity is highest in the liver, intermediate in kidneys and heart, and low in muscles, adipose tissue, and brain [3]. When the weights of individual tissues are taken into consideration, muscles, which make up 35 to 40% of total body weight, should contribute substantially to total body BCAA utilization. Thus, BCAA degradation is under joint control of a number of tissues, among which the muscle and liver play a dominant role (Fig. 2). Many influences including cytokines, hormones, nutrients, and various metabolites affect the activity state of the enzyme [3]. The remarkable rise in BCKD activity in muscles induces endotoxin or tumor necrosis factor alpha (TNF-α) administration [4, 5].

Beyond the BCKD reaction, the metabolism of the BCAA diverges into separate pathways. Catabolism of KIC leads to acetyl-CoA and acetoacetate (KIC is ketogenic), KIV is catabolized to succinyl-CoA (KIV is glucogenic), and KMV to acetyl-CoA and succinyl-CoA (KMV is both glycogenic and ketogenic). A special product of KIC catabolism is β-hydroxy-β-methylbutyrate (HMB) synthesized in the reaction catalyzed by KIC dioxygenase.

### Amination of the BCKA and interorgan cycling of the BCAA and BCKA (Fig. 3)

As the BCAT reaction is reversible and near equilibrium, its direction should respond to changes in concentrations of BCAA and BCKA, and availability of the donors and acceptors of nitrogen. In most conditions the majority of the BCAA uptake and BCKA release occurs in muscles, while amination of the BCKA to the BCAA may occur in other tissues, notably in the liver, kidneys and enterocytes. Direct evidence of the BCKA amination to the BCAAs was provided by studies using labelled BCKA showing the labelled BCAA in proteins [6]. The main sources of nitrogen for amination of the BCKA are GLN, glutamate, and ALA [3].

**Fig. 3:**
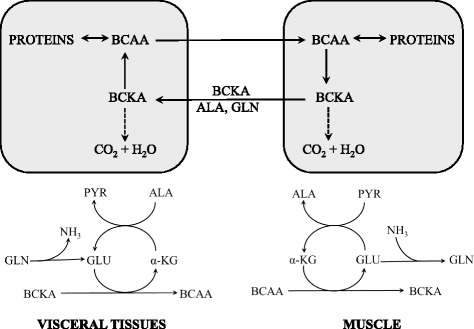
The schemes of the BCAT reactions (BCAA deamination and BCKA amination) and supposed cycling of the BCAA and BCKA among organs, which may in various conditions attenuate the loss of essential BCAA [11]. ALA, alanine; BCAA, branched-chain amino acids; BCKA, branched-chain keto acids. GLU, glutamate; GLN, glutamine; PYR, pyruvate; α-KG, α-ketoglutarate

The studies demonstrate that BCAA synthesis from the BCKA is activated in various muscle wasting disorders, e.g. sepsis, trauma, or surgery, in which the muscles release high amounts of GLN and ALA to the blood [7–11]. A marked increase in leucine release was observed by the isolated liver of endotoxin-treated animals after addition of KIC into perfusion medium [7]; higher synthesis of the BCAA from BCKA was shown in the liver perfused with medium containing 0.5 mM GLN when compared to perfusion with medium without GLN [8]. Amination of the BCKA may have a role in the unique increase of all three BCAA in blood plasma during a brief starvation characterized by accelerated release of ALA, GLN, and BCKA from muscles and augmented gluconeogenesis in the liver [9, 10].

The above-mentioned findings indicate the existence of an interorgan cycle (Fig. 3) that attenuates the loss of essential BCAA in various physiological and pathological conditions [11].

## Functions of the BCAA

**Fig. 4:**
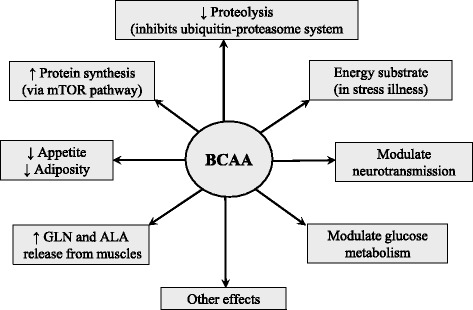
Supposed effects of BCAA supplementation. ALA, alanine; BCAA, branched-chain amino acids; GLN, glutamine; ↑, increase; ↓, decrease

The BCAAs serve as substrates for protein synthesis or energy production and perform several metabolic and signaling functions, particularly via activation of the mammalian target of rapamycin (mTOR) signaling pathway. The following roles of the BCAA should be considered as crucial for their use as nutritional supplements (Fig. 4).

### Effects on protein metabolism

BCAAs not only serve as substrates for protein synthesis, but also exert stimulatory effect on protein synthesis and an inhibitory effect on proteolysis. The effects are realized by the BCAAs themselves, especially by leucine, and their metabolites. Leucine stimulates protein synthesis through the mTOR signaling pathway and phosphorylation of translation initiation factors and ribosomal proteins [12]. A role in protein anabolic effect of leucine plays also its stimulatory effect on insulin secretion [13]. The inhibitory effect of the BCAA on proteolysis is mediated mainly by BCKAs and HMB. BCKAs have been shown to prevent proteolysis in muscles under in vitro conditions [14]. Infusions of KIC were more effective than leucine in maintaining nitrogen balance in fasted subjects and in patients undergoing major abdominal surgery [15, 16]. HMB decreases the activity of the ubiquitin-proteasome proteolytic pathway and exerts beneficial effects on muscle in various conditions of health and disease [17].

### Effects on neurotransmission

BCAAs are transported into the brain via the same carrier that transports aromatic amino acids (AAA; phenylalanine, tyrosine, tryptophan), and competition between BCAAs and AAAs may influence synthesis of some neurotransmitters, notably dopamine, norepinephrine, and 5-hydroxytryptamine (serotonin). Therefore, elevation of the BCAA in blood plasma is able to influence neurotransmitter levels in the brain with effects on behavior and brain function. This phenomenon is the rationale for use of the BCAAs in patients with liver cirrhosis, in which a decreased ratio of BCAAs to AAAs plays a role in pathogenesis of hepatic encephalopathy [18]. It is believed that BCAA supplementation attenuates production of serotonin, which is responsible for fatigue during exercise. Furthermore, BCAA transamination in the brain plays a role in the synthesis of glutamate and gamma-aminobutyric acid, and in ammonia detoxification to GLN in astrocytes. The studies have shown that leucine decreases appetite and may decrease body adiposity [19].

### Effects on glucose metabolism

There are close associations between BCAAs and plasma glucose levels. The fact that BCAAs upregulate glucose transporters and activate insulin secretion has been widely demonstrated [13, 20, 21]. However, several researchers have suggested that excessive intake of amino acids could lead to inhibition of insulin signaling [22, 23]. Recent studies have suggested differential effects of each BCAA on glucose utilization and that BCAAs may induce insulin resistance through mTOR activation [24]. Further investigation is needed to understand variable reports ranging from improving glucose utilization to inducing insulin resistance.

### Effects mediated by ALA and GLN

The rate of BCAA degradation in skeletal muscle is highly responsive to their availability [25]. The consequences of this phenomenon are that the primary effects of the consumption of a BCAA-enriched diet are activated catabolism of the BCAAs and enhanced levels of the BCKAs, ALA, and GLN in peripheral circulation [26]. Therefore, a number of effects of BCAA supplementation are mediated by ALA and GLN. ALA is the main gluconeogenic amino acid, and GLN availability is essential for immune system, glutathione production, maintenance of acid-base balance by the kidneys, and expression of heat shock proteins.

### Other effects

During recent years, a number of novel functions of BCAAs, including benefits for mammary health and milk quality, intestinal development, immune response, mitochondrial biogenesis and oxidative stress have been reported [21].

## Effects of starvation and diets with a different protein content on metabolism and BCAA levels

BCAA metabolism is very sensitive to changes in the amount and composition of the food, which may occur in both healthy and disease states. Here, I have attempted to explain the effects of starvation and diets with low and high protein contents.

### Starvation

Brief starvation uniquely increases BCAA concentrations in plasma. In humans, the increase is evident within a day, and reaches maximum by the second or third day [27, 28].

Both increased proteolysis and reduced protein synthesis in muscles have been reported during brief starvation and may explain the enhanced availability of BCAAs for muscles [10, 29, 30]. In this condition, BCAAs in muscles act as a source of nitrogen for synthesis of ALA and GLN, which are released into the blood and used in visceral tissues, especially as gluconeogenic substrates. Increased BCAT activity in muscles during starvation has been reported by several laboratories [31, 32].

Together with ALA and GLN, the BCKAs generated in BCAT reaction are released into circulation and their concentration in the blood increases [26]. It may be supposed that a portion of nitrogen released during catabolism of GLN and ALA in visceral tissues escapes utilization in the urea cycle and is used for amination of BCKAs. Higher rates of BCAA synthesis from the BCKAs were observed by the liver perfused with GLN-containing medium than that perfused with GLN-deficient medium [8]. A role in the increase of BCAAs may have also their decreased uptake from the blood due to the decreased levels of insulin. An unresolved possibility is the activated breakdown of proteins in the liver, which may, due to low activity of hepatic BCAT, result in the release of the BCAA into the blood.

Prolonged starvation lowers the BCAA concentration to basal levels and gradually increases the activity of the BCKD complex. Marked increase in BCKD activity in muscles and heart occurs in the terminal phase of starvation, when amino acids replace fatty acids and ketone bodies as the predominant energy substrate [33].

### Effects of a low-protein diet

Feeding healthy human volunteers or animals a diet devoid of protein, but adequate in caloric content, lowered the plasma BCAA concentrations below basal levels [27, 34]. The amino acid pattern of children with severe kwashiorkor shows severe decrease of BCAAs [35].

It is believed that the principal factors in the decrease of BCAAs during protein deprivation are the absence of exogenous amino acids as well as curtailed muscle protein breakdown. Lowered BCKD activities in muscles and liver of protein-depleted rats indicate the effort of the body to conserve BCAAs [36].

BCAA or BCKA supplementation should be recommended when a low-protein diet is prescribed to patients with chronic renal failure or urea cycle disorders.

### Effects of a high-protein diet

Increased intake of protein may increase protein synthesis, decrease protein breakdown, reduce fat accumulation, and increase fat-free mass. Therefore protein supplementation or a high-protein diet is recommended to build the muscles in athletes, to prevent muscle wasting in severe illness, and to lose fat in the treatment of obesity.

High concentrations of BCAAs and urea are found in the postprandial state in the peripheral blood and muscles after intake of a protein meal and in subjects consuming a high-protein diet. In contrast to increased BCAA levels, the increments in arterial concentrations of most remaining amino acids of the ingested protein are small or insignificant [37, 38].

The main cause of the specific BCAA increase is the unique distribution of the enzymes, which control BCAA catabolism. While complete oxidation of most individual amino acids occurs in the liver, the initial site of BCAA catabolism is skeletal muscle. Therefore, a significant portion of ingested BCAA escapes hepatic uptake and appears in peripheral circulation. The effects of protein ingestion on BCAA levels are not observed in a postabsorptive state [38].

## Disorders with decreased BCAA levels

The studies have shown that BCAA deficiency impairs mRNA translation and dietary inadequacies of BCAA result in impaired growth and protein wasting [12, 39, 40]. In addition, studies in human subjects have shown that decreased BCAA level may influence synthesis of neurotransmitters and adversely affect brain function [18, 41]. Therefore, BCAA supplementation seems rational in disorders with decreased BCAA levels, which occur in liver cirrhosis, urea cycle disorders, and chronic renal insufficiency.

### Liver cirrhosis

**Fig. 5:**
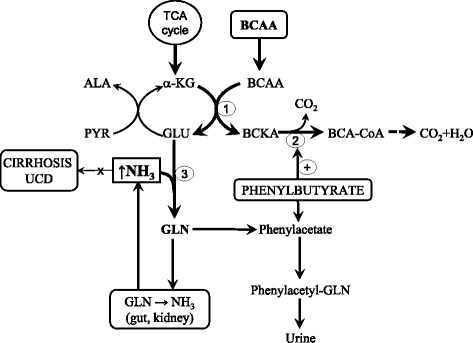
Pathways of ammonia detoxification to GLN in muscles associated with enhanced consumption of the BCAA and α-KG (cataplerosis) and suggested effects of BCAA and phenylbutyrate in subjects with liver cirrhosis or UCD. Positive effect of BCAA on ammonia detoxification to GLN may be blunted by GLN degradation to ammonia in enterocytes and kidneys. Phenylbutyrate decreases ammonia via enhanced excretion of GLN by urine. An adverse side effect of phenylbutyrate is activation of BCKD resulting in the decrease of the BCAA. ALA, alanine; BCAA, branched-chain amino acids; BCKA, branched-chain keto acids. GLU, glutamate; GLN, glutamine; PYR, pyruvate; TCA cycle, tricarboxylic acid cycle; UCD, urea cycle disorders; α-KG, α-ketoglutarate. 1, branched-chain-amino-acid aminotransferase; 2, branched-chain α-keto acid dehydrogenase; 3, GLN synthetase

The decrease in BCAAs and an increase in AAAs are characteristic alterations in the blood of subjects with liver cirrhosis, which play a role in pathogenesis of hepatic encephalopathy and muscle wasting [18, 42]. Several studies have shown an inverse relationship between plasma ammonia and BCAA concentrations in patients with cirrhosis and that ammonia infusion decreases BCAA levels [43, 44]. BCAAs decrease because they are rapidly consumed to form glutamate from α-KG as a pivotal step in ammonia detoxification to GLN in muscles and in the brain [45]. Accelerated consumption of α-KG (cataplerosis) may disturb the function of the tricarboxylic acid (TCA) cycle (Fig. 5). The AAA increase is due to the decreased ability of the diseased liver to metabolize these amino acids. The BCAA levels do not decrease in acute liver injury due the leaking of amino acids from dying hepatocytes into the circulatory system [46].

#### Effects of BCAA supplementation

BCAAs are recommended to ameliorate cachexia and the decreased ratio of BCAAs to AAAs, which plays a role in the pathogenesis of hepatic encephalopathy. Potential benefits also include positive effects of the BCAA on ammonia detoxification to GLN in muscles, liver regeneration, albumin synthesis, immune and hepatic function, glucose metabolism, and physical and mental fatigue [20, 47–49].

Unfortunately, the results from clinical trials do not provide strong evidence of their beneficial effects [50, 51] and adverse effects of BCAA supplementation, which may compete with their benefits, have also been suggested [52]. The positive effects of BCAAs in subjects with liver cirrhosis may be blunted by enhanced catabolism of GLN produced in muscles to ammonia in visceral tissues, especially in the gut and kidneys. The draining of α-KG from TCA cycle may also be detrimental (Fig. 5). Therefore, therapeutic strategies are needed to avoid potential adverse effects of BCAAs on ammonia production and cataplerosis. Options include substitution of α-KG, glutamate related substrates (e.g. L-ornithine-L-aspartate), GLN elimination from the body by phenylbutyrate, replacement of BCAAs by BCKAs, and optimizing dose, proportions, and timing of BCAA supplementation [52].

### Urea cycle disorders (UCD) and phenylbutyrate

UCD result from inherited enzymatic defects in the ammonia detoxification pathway in the liver, leading to low levels of urea and high levels of ammonia in the blood. The disorders are characterized by seizures, lethargy, coma, and death in the neonatal period or severe long-term neurological impairment.

In addition to altered levels of ammonia and urea, common finding in patients with UCD is an increase in GLN and a decline in BCAA levels, notably during acute metabolic decompensation [53]. These alterations support the theory that BCAAs play a unique role in ammonia detoxification to GLN and that hyperammonemia is the cause of decreased BCAA levels in subject with liver cirrhosis [45, 54].

At present, the management of UCD is achieved by dietary protein restriction and the use of compounds that remove nitrogen, notably benzoate and phenylbutyrate. Benzoate conjugates glycine to promote the synthesis of hippuric acid that is eliminated in urine and thus attenuates catabolism of glycine to ammonia. Phenylbutyrate is converted by β-oxidation into phenylacetate that is conjugated with GLN to form phenacetylglutamine, which is excreted in the urine (Fig. 4). Unfortunately, it has been shown that phenylbutyrate activates the BCKD, resulting in decreases in BCAA and BCKA levels in blood plasma [55, 56]. Marked decrease of BCAAs in UCD after phenylbutyrate treatment has been reported by Scaglia et al. [57].

#### Effects of BCAA supplementation

Low BCAA levels in subjects with UCD, especially those treated by phenylbutyrate, indicate the rationale to use BCAAs as a therapeutic agent. Unfortunately, the reports of attempts to use BCAAs in UCD are unique. Cross-sectional data from 41 European Inherited Metabolic Disorder centers reported that only 16 (3%) patients (from 8 centers in 5 countries) received BCAA supplements. The two most common conditions were ornithine transcarbamylase deficiency and citrullinaemia [58].

### Chronic renal failure (CRF)

Most studies of amino acid patterns in CRF reported decreased BCAA and BCKA levels in the blood plasma [59–61] and reduced concentrations of valine in muscles [61, 62]. The derangements are caused by the action of multiple factors, notably acidosis and glucocorticoids. Decreased intake of proteins and hemodialysis, resulting in low concentrations of most essential and nonessential amino acids, is also a factor. In contrast to CRF, inconsistent alterations have been reported in acute renal failure.

Several articles have suggested that metabolic acidosis is responsible for accelerated proteolysis and enhanced activity of the BCKD in muscles and liver [63, 64]. More significant increases in proteolysis and leucine oxidation were reported in rats with chronic uremia and acidosis when compared with uremic rats without acidosis. A significant decrease in valine concentration in the gastrocnemius muscle was found only in rats with acidosis [61].

#### Effects of BCAA supplementation

BCAAs and BCKAs are supplied to patients with CRF together with other essential amino acids and their ketoanalogues to decrease protein intake as much as possible to maintain protein balance and avoid its deleterious effects on urea levels [65, 66].

## Disorders with enhanced BCAA levels

Increased BCAA concentrations are found in various insulin-deficient and -resistant states, especially diabetes and obesity. Very high BCAA and BCKA concentrations are found in maple syrup urine disease (MSUD).

### Type 1 diabetes

High BCAA levels in subjects with defective insulin secretion were first described in dogs with experimental diabetes [67]. Further studies have shown that in addition to the increase of BCAAs, there is a decrease in levels of gluconeogenic amino acids, especially ALA [68–70]. Most data on pathogenesis of high levels of the BCAA in diabetes type 1 originate from studies using animals with diabetes induced by streptozotocin or alloxan.

There are some similarities in the pathogenesis of the increased BCAAs in diabetes and short-term starvation, which is also an insulin deficient state. As in starvation, a role play activated amination of the BCKAs in the liver and impaired uptake of the BCAA by muscles. The BCKA levels increase in blood plasma and muscles of rats with chemically-induced diabetes, but decline in the liver [71]. The role of the liver as a source of BCAAs is supported by observations of reduced activity of hepatic BCKD in rats with severe ketotic diabetes [72].

However, unlike brief starvation, the changes in diabetes are associated with marked increase in proteolysis and BCKD activity in muscles, resulting in severe cachexia [73]. While muscle nitrogen repletion occurs and BCAA levels are normalized after feeding of previously starving subjects, the BCAAs accumulate and diminished nitrogen repletion remains after feeding in subjects with type 1 diabetes [74].

### Obesity and type 2 diabetes

Plasma concentrations of BCAAs are frequently elevated in obesity and type 2 diabetes [75–77]. The mechanism responsible for the increased BCAAs in these insulin-resistant states is not completely clear. A major cause might be reduced activity of the BCKA dehydrogenase, which was reported in the liver and adipose tissue in genetically obese ob/ob mice, Zucker rats and spontaneous type 2 diabetes Otsuka Long-Evans Tokushima Fatty (OLETF) rats [76, 77].

The studies have shown that the BCAA levels in obesity correlate with insulin resistance and are a sensitive predictor of diabetes in the future [78, 79]. Recent studies have suggested that high levels of the BCAA interfere with oxidation of fatty acids in muscles, leading to accumulation of various acylcarnitines and insulin resistance [24].

#### BCAA supplementation

Conflicting results have been reported concerning the effects of BCAA supplementation in subjects with insulin resistance. Leucine improved glucose tolerance, decreased hepatic steatosis, and decreased inflammation in adipose tissue in mice fad a high-fat diet [80] and rescued insulin signaling in adipose tissue obtained from insulin resistant db/db mice [81]. Arakawa et al. [82] reported that BCAAs reduced hepatic and triglyceride concentrations in mice fed a high-fat diet. On the other hand, Newgard et al. [79] showed that administration of a mixture of BCAA to rats on a high-fat diet increased insulin resistance. White et al. [24] demonstrated that the BCAA-restricted diet improved muscle insulin sensitivity in Zucker-fatty rats.

### Maple syrup urine disease (MSUD)

MSUD is recessive disorder caused by a severe deficiency of BCKD activity. All three BCAAs, as well as the corresponding BCKAs, are elevated in blood, tissues, and urine. High BCAA and BCKA levels are related to excitotoxicity, energy deficit, and oxidative stress in the brain, resulting in severe neurological symptoms.

#### BCAA supplementation

BCAA administration to subjects with MSUD is inappropriate. DNA damage in the hippocampus and the striatum was demonstrated after administration of BCAAs in an animal model of MSUD [83]. Current treatment of MSUD is based on protein restriction and synthetic formulas with reduced BCAA content. Perspective may be phenylbutyrate, which activates BCKD and decreases BCAA and BCKA levels [55, 56]. Unfortunately, studies examining phenylbutyrate in MSUD patients are unique. Decreased BCAA and BCKA levels were reported in three out of the five MSUD patients treated by phenylbutyrate (10 g/m^2^) for one day [56]. Long-term studies in different MSUD phenotypes are indicated to verify phenylbutyrate efficacy.

## Conditions with enhanced BCAA catabolism and inconsistent alterations in BCAA levels

### Exercise

Physical exercise is associated with enhanced BCAA oxidation and GLN release from muscles [84, 85]. Evidence suggests that BCKD is activated by dephosphorylation mediated by falling ATP levels within the muscles during exercise. Training appears to increase mRNA expression of this enzyme [86]. The plasma BCAA levels during or after exercise have been reported to be unchanged [87], to decrease [88], or to increase [89]. The cause of inconsistent response can be explained by different work load and duration of exercise.

#### Effects of BCAA supplementation

BCAAs are recognized as supplements for athletes with a number of benefits, notably on muscle protein synthesis, fatigue recovery, and exercise-induced muscle damage [90]. In addition to the positive reports, there are a number of reports showing no benefits of BCAA supplementation [91]. Of special interest should be findings of enhanced blood ammonia levels after BCAA administration during exercise suggesting that exogenous BCAA may exert negative effects on muscle performance via ammonia [92, 93]. Additional studies are needed to assess the true efficacy of BCAA supplementation on muscle performance and fatigue.

### Hypermetabolic states accompanied by systemic inflammatory response syndrome

**Fig. 6:**
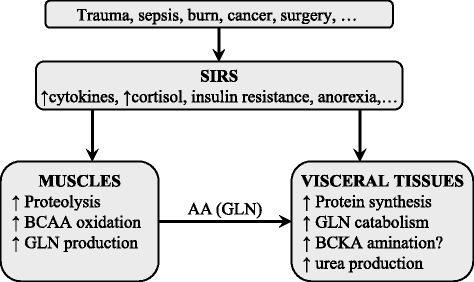
Main alterations in protein and BCAA metabolism in disorders accompanied by SIRS. AA, amino acids; BCAA, branched-chain amino acids; BCKA, branched-chain keto acids; GLN, glutamine; SIRS, systemic inflammatory response syndrome; ↑, increase; ↓, decrease

There are several hypermetabolic states (e.g. sepsis, burn injury, trauma, and cancer) in which alterations in BCAA levels are not consistent, with increased, unchanged, and decreased levels being reported. Present in all of these conditions is systemic inflammatory response syndrome (SIRS) characterized by a wide range of neuro-humoral abnormalities, including enhanced production of cytokines, sympathetic nervous system activation, and cortisol production. These events cause several alterations in metabolism, including insulin resistance and enhanced myofibrillar protein degradation, resulting in severe depletion of lean body mass. If the hypermetabolic state persists, multisystem organ failure and eventually death may occur (Fig. 6).

In this situation, BCAAs act as a significant energy substrate for muscles [4, 5, 94]. Increased BCAA oxidation is coupled with increased synthesis of GLN, which is released from muscles and utilized, preferably by the immune system. Utilization of GLN often exceeds its synthesis, leading to a lack of GLN in blood and tissues [95, 96]. Decreased GLN availability can become rate-limiting for key functions of immune cells, such as phagocytosis and antibody production. Decreased GLN levels have been shown to act as a driving force for BCAA utilization in muscles [97]. Studies have also indicated that inflammatory signals decrease BCAA absorption from the gut and inhibit BCAA transport from the blood to muscles, while promoting transport into the liver [98, 99]. The BCAA synthesis from the BCKA in visceral tissues is probably activated. A marked increase in leucine release was observed by the isolated liver of endotoxin-treated animals after the addition of KIC into perfusion medium [7].

The cause of inconsistent alterations in BCAA levels although their oxidation is remarkably activated are different influences of individual metabolic changes occurring in the SIRS. Increased protein breakdown or decreased protein synthesis in muscles and insulin resistance may enhance the BCAA levels. Activation of BCAA catabolism associated with enhanced ALA and GLN production in muscles and protein synthesis in visceral tissues decrease the BCAA levels. Therefore, alterations in BCAA levels are inconsistent.

#### BCAA supplementation in burn, trauma, and sepsis

Rationales for the use of BCAA supplements in conditions with SIRS are their enhanced oxidation, which may limit their availability in tissues and their protein anabolic properties. Benefits of BCAAs may also be related to their role as a precursor of GLN, which is a key factor in maintaining immune functions and gut integrity, and has a favorable influence on protein balance.

Various solutions containing different amounts and proportions of individual BCAA have been used to examine their effects in trauma, burn, or sepsis. A number of investigators have reported that BCAA ameliorate negative nitrogen balance [100–102]. However, the results of other investigators have not been impressive, and there is no scientific consensus regarding the effect BCAA-enriched formulas on protein balance, length of hospital stay, and mortality [103–105]. A serious shortcoming of most of the studies is the lack of information regarding BCAA concentrations in blood and tissues, which may be suggested as a possible criterion of eligibility of the indication.

The low effectiveness of the BCAA in disorders with the presence of SIRS may be related to insulin resistance and metabolic alteration associated with inflammation. Studies have shown that inflammatory response blunts the anabolic response to BCAA administration. Lang and Frost [106] demonstrated that leucine induced activation of eukaryotic initiation factor eIF4E is abrogated in endotoxin-treated rats and that endotoxin treatment antagonized the leucine-induced phosphorylation of ribosomal protein S6 and mTOR.

In recent years articles have emerged suggesting positive effects of BCAA in traumatic brain injury. In rodents, BCAAs have demonstrated to ameliorate injury-induced cognitive impairment [107], and clinical studies have demonstrated that BCAAs enhance the cognitive recovery in patients with severe traumatic brain injury [108, 109].

#### BCAA supplementation in cancer

Unlike other states accompanied by SIRS, muscle wasting and amino acid mobilization from muscles in subjects with cancer may be driven by secretion of different tumor-derived mediators. Therefore, progressive depletion of muscle mass may be observed in some cancer patients. Also high rates of BCAA oxidation in muscles of subjects with cancer have been reported [110]. Increasing evidence demonstrates that BCAAs are essential nutrients for cancer growth and are used as a source of energy by tumors. Expression of the cytosolic type of BCAT has been shown to correlate with more aggressive cancer growth [111].

The findings of clinical trials examining the effects of BCAA-enriched nutritional support to cancer patients are inconsistent. Some showed improved nitrogen balance and reduced skeletal muscle catabolism whereas others show no significant improvement [112]. A concern in the tumor-bearing state is that provision of the BCAA will promote tumor growth.

## Summary and conclusions

The studies indicate that important role in pathogenesis of alterations in BCAA metabolism play: (i) skeletal muscle as initial site of BCAA catabolism accompanied by the release of GLN, ALA, and BCKA to the blood; (ii) activity of BCKD in muscles and liver, and (iii) amination of BCKA to corresponding BCAA, especially by nitrogen of ALA and GLN released from muscles. Here are examples of importance of these metabolic steps:

ad (i) Because the muscle is the initial site of BCAA catabolism, marked rise of BCAA is observed after a meal while the rise of other amino acids is small. Enhanced consumption of the BCAA for ammonia detoxification to GLN in muscles is the main cause of the decrease of the BCAA in hyperammonemic conditions (liver cirrhosis, UCD). Increased production of GLN after BCAA intake in muscles may lead to enhanced production of ammonia in enterocytes and kidneys with deleterious effect in subjects with liver disease.

ad (ii) Decreased BCKD activity is the main cause of increased BCAA and BCKA levels in MSUD and may play a role in increased BCAA levels in obesity and type 2 diabetes. Increased BCKD activity is responsible for the decrease of BCAAs in CRF and enhanced oxidation of BCAAs during exercise and in various hypermetabolic conditions (burn, sepsis, trauma, cancer).

ad (iii) BCKA amination partially explains the increased BCAA concentrations during brief starvation and in type 1 diabetes, and is the basis of rationale to use BCKA-enriched supplements in CRF therapy.

Although amino acid concentrations in the plasma pool are poor indicators of their requirements, it may be suggested that under conditions of good understanding of the BCAA metabolism in specific disorder, the BCAA levels would conceptually be an acceptable argument for their supplementation. It may be supposed that:

1. Together with requirements to decrease protein content in a diet, increased oxidation and low BCAA levels are a clear rationale to use the BCAA together with other essential amino acids and their ketoanalogues in CRF therapy.
2. Although BCAA decrease in blood plasma is a rationale to use the BCAA supplements in patients with liver cirrhosis and UCD, therapeutic strategies are needed to avoid detrimental effects of BCAA supplementation on ammonia production.
3. Further studies are necessary to conclude the question of the effects of BCAA supplementation in burn, trauma, sepsis, cancer, and exercise. A very small number of clinical studies have reported the effects of BCAA supplementation in relation to amino acid concentrations in blood and tissues.
4. Whether increased BCAA levels only markers are or also contribute to insulin resistance should be known before the decision is taken regarding their suitability in obese subjects and patients with type 2 diabetes.

In conclusion, alterations in BCAA metabolism are common in a number of disease states and the BCAA have therapeutic potential due to their proven protein anabolic effects. However, many controversies about the use of BCAAs in clinical practice still exist, and careful studies are needed to elucidate the effectiveness of BCAAs in most indications.

## Abbreviations

KIC: α-ketoisocaproate (ketoleucine)
KIV: α-ketoisovalerate (ketovaline)
KMV: α-keto-β-methylvalerate (ketoisoleucine)
ALA: Alanine
BCAA: Branched-chain amino acids
BCAT: Branched-chain-amino-acid aminotransferase
BCKA: Branched-chain keto acids
BCKD: Branched-chain α-keto acid dehydrogenase
CRF: Chronic renal failure
GLN: Glutamine
MSUD: Maple syrup urine disease
SIRS: Systemic inflammatory response syndrome
TCA cycle: Tricarboxylic acid cycle
UCD: Urea cycle disorders; α-KG, α-ketoglutarate
